# Attitudes and Use of Information and Communication Technologies in Older Adults With Mild Cognitive Impairment or Early Stages of Dementia and Their Caregivers: Cross-Sectional Study

**DOI:** 10.2196/17253

**Published:** 2020-06-01

**Authors:** Jose Guzman-Parra, Pilar Barnestein-Fonseca, Gloria Guerrero-Pertiñez, Peter Anderberg, Luis Jimenez-Fernandez, Esperanza Valero-Moreno, Jessica Marian Goodman-Casanova, Antonio Cuesta-Vargas, Maite Garolera, Maria Quintana, Rebeca I García-Betances, Evi Lemmens, Johan Sanmartin Berglund, Fermin Mayoral-Cleries

**Affiliations:** 1 Mental Health Department Instituto de Investigación Biomédica de Málaga University Regional Hospital of Malaga Malaga Spain; 2 Department of Health Blekinge Institute of Technology Karlskrona Sweden; 3 Departamento de Fisioterapia Instituto de Biomedicina de Málaga Universidad de Málaga Malaga Spain; 4 Brain, Cognition and Behavior – Clinical Research Consorci Sanitari de Terrassa Barcelona Spain; 5 Life Supporting Technologies Group Universidad Politécnica Madrid Spain; 6 University Colleges Leuven-Limburg Genk Belgium

**Keywords:** aging, mild cognitive impairment, dementia eHealth, information and communication technology, technophilia

## Abstract

**Background:**

Information and communication technologies are promising tools to increase the quality of life of people with dementia or mild cognitive impairment and that of their caregivers. However, there are barriers to their use associated with sociodemographic factors and negative attitudes, as well as inadequate knowledge about technologies.

**Objective:**

The aim of this study was to analyze technophilia (attitudes toward new technologies) and the use of smartphones and tablets along with associated factors in people with dementia/mild cognitive impairment and their caregivers.

**Methods:**

Data from the first visit of the Support Monitoring and Reminder for Mild Dementia (SMART4MD) randomized multicenter clinical trial were used for this analysis. Data were obtained from two European countries, Spain and Sweden, and from three centers: Consorci Sanitari de Terrassa (Catalonia, Spain), Servicio Andaluz de Salud (Andalusia, Spain), and the Blekinge Institute of Technology (Sweden). Participants with a score between 20 and 28 in the Mini Mental State Examination, with memory problems (for more than 6 months), and who were over the age of 55 years were included in the study, along with their caregivers. The bivariate Chi square and Mann-Whitney tests, and multivariate linear and logistic regression models were used for statistical analysis.

**Results:**

A total of 1086 dyads were included (N=2172). Overall, 299 (27.53%) of people with dementia/mild cognitive impairment had a diagnosis of dementia. In addition, 588 (54.14%) of people with dementia/mild cognitive impairment reported using a smartphone almost every day, and 106 (9.76%) used specific apps or software to support their memory. Among the caregivers, 839 (77.26%) used smartphones and tablets almost every day, and 181 (16.67%) used specific apps or software to support their memory. The people with dementia/mild cognitive impairment showed a lower level of technophilia in comparison to that of their caregivers after adjusting for confounders (B=0.074, *P*=.02) with differences in technology enthusiasm (B=0.360, *P*<.001), but not in technology anxiety (B=–0.042, *P*=.37). Technophilia was associated with lower age (B=–0.009, *P*=.004), male gender (B=–0.160, *P*<.001), higher education level (*P*=.01), living arrangement (living with children vs single; B=–2.538, *P*=.01), country of residence (Sweden vs Spain; B=0.256, *P*<.001), lower depression (B=–0.046, *P*<.001), and better health status (B=0.004, *P*<.001) in people with dementia/mild cognitive impairment. Among caregivers, technophilia was associated with comparable sociodemographic factors (except for living arrangement), along with a lower caregiver burden (B=–0.005, *P*=.04) and better quality of life (B=0.348, *P*<.001).

**Conclusions:**

Technophilia was associated with a better quality of life and sociodemographic variables in people with dementia/mild cognitive impairment and caregivers, suggesting potential barriers for technological interventions. People with dementia/mild cognitive impairment frequently use smartphones and tablets, but the use of specific apps or software to support memory is limited. Interventions using these technologies are needed to overcome barriers in this population related to sociodemographic characteristics and the lack of enthusiasm for new technologies.

**Trial Registration:**

ClinicalTrials.gov NCT03325699; https://clinicaltrials.gov/ct2/show/NCT03325699

## Introduction

The population in Europe is getting older, and consequently the number of people with dementia or mild cognitive impairment is increasing given the association of these conditions with age. It is estimated that the population with dementia will double by 2030 and triple by 2050, reaching more than 115 million individuals [1]. One of the core symptoms in mild cognitive impairment and in the early stages of dementia is memory impairment, which is a condition that is also associated with depression, sleep problems, and other behavioral symptoms [2,3]. Guaranteeing an optimal quality of life for people with these conditions remains an enormous challenge because there are no effective long-term pharmacological treatments in the majority of cases [4].

Information and communication technologies (ICTs), especially touchscreen technologies, are promising tools to increase the quality of life and cognitive function of people with dementia/mild cognitive impairment and their caregivers [5-9]. These technologies could be used to train cognitive functions, monitor health and movements, provide reminders to support memory, promote social support, improve communication with caregivers, and provide useful information about the condition. Smartphones and tablets have the advantage of not raising a stigma for the individuals that use them [10] because they are ubiquitous and used by the majority of the population, and they also represent a natural source of data for professionals and researchers [10-13]. Over 40,000 health-related apps exist but very few are specifically designed for people with dementia/mild cognitive impairment [14]. Previous studies have found barriers associated with the use of ICTs in older adults, including age and education level [15]. Likewise, barriers for the use of technology in people with dementia/mild cognitive impairment have been described, including negative attitudes toward ICTs, inaccurate perceptions of ICTs, and poor technology knowledge [16]. Although the attitudes and knowledge of ICTs and touchscreen technologies could determine the use of health apps, to the best of our knowledge, few large-sample studies  have investigated the attitude and use of these technologies in older adults, including people with dementia/mild cognitive impairment and their caregivers. Technophilia is one of the emerging concepts regarding attitudes toward technologies. One of the definitions of technophilia is the “attraction, enthusiasm of the human individual determined by the activities which involve the use of advanced technologies. It is expressed by easy adaptation to the social changes brought by technological innovations” [17].

The aim of this study was to analyze technophilia in people with dementia/mild cognitive impairment and their caregivers and to determine the sociodemographic and clinical factors associated with technophilia. Another aim was to analyze how this population uses smartphones, tablets, apps, and software to support their memory, and to identify factors associated with the use of apps to support memory. We tested the hypothesis that different sociodemographic and clinical factors in people with dementia/mild cognitive impairment and their caregivers are associated with technophilia and the use of specific apps to support memory in tablets or smartphones.

## Methods

### Study Design

In this cross-sectional study, data from the baseline assessment of the Support Monitoring and Reminder for Mild Dementia (SMART4MD) randomized multicenter clinical trial (ClinicalTrials.gov NCT03325699) were used. The objective of the trial was to create a digital platform (SMART4MD) for a tablet and to test if the platform had an impact on the quality of life of people with dementia/mild cognitive impairment and their caregivers. More detailed information on the trial is available in the published protocol [18].

### Setting

This study was carried out in two European countries, Spain and Sweden, and at three centers: Consorci Sanitari de Terrassa (Catalonia, Spain), Servicio Andaluz de Salud (Andalusia, Spain), and the Blekinge Institute of Technology (Sweden).

### Participants

A total of 1086 participant dyads (N=2172) were included in the study. The participant dyads comprised people with dementia/mild cognitive impairment and their informal caregivers. The participants were selected using a nonprobabilistic consecutive sampling method. The inclusion criteria were as follows: (1) score of 20 to 28 points on the Mini Mental State Examination (MMSE), (2) experience of memory problems over a substantial period of time (more than 6 months), (3) aged>55 years, (4) recipients of home care, (5) have an informal caregiver, (6) taking prescribed medication and in charge of it, and (7) no physical conditions that would reduce their ability to use a touchscreen app. The exclusion criteria were as follows: (1) terminal illness with less than 3 years of expected survival, (2) score above 11 on the Geriatric Depression Scale (GDS-15), or (3) another known significant cause of disease as an explanation for cognitive impairment such as substance abuse, bipolar disorder, schizophrenia, or developmental disorders.

### Measures

#### Dependent Variables

The dependent variables were technophilia and use and familiarity with touchscreen devices. The TechPH questionnaire was used to assess technophilia [19]. This questionnaire includes 6 items assessed on a 5-point Likert scale from 1 (fully disagree) to 5 (fully agree), which was designed to specifically assess technophilia in the older population. The scale has two factors: technology enthusiasm and technology anxiety. The TechPH index is a score derived from the 6 items (the sum of items divided by 6) ranging from 1 to 5. More information on the TechPH index is included in the report on the validation study [19].

Use and familiarity with touchscreen devices was assessed with a questionnaire tailored to this study covering the following aspects: (1) use of a smartphone and tablets (“On average how often would you say you have been using a smartphone or tablet during the last 3 months?”), (2) use of the internet (“How often do you use the internet on your smartphone or tablet?”), (3) knowledge (“How knowledgeable do you consider yourself when it comes to using a smartphone or a tablet?”), (4) use of technology to support memory (“Are you using your mobile phone or tablet as a way to support your memory today?”), (5) use of specific apps for memory (“Do you have any special app or software on your mobile phone or tablet that you use to support your memory?”), and (6) perspective on the helpfulness of the technology for their memory (“Do you think that using your mobile phone or tablet to support your memory helps you to remember things?”).

#### Independent Variables in People With Dementia/Mild Cognitive Impairment

Health-related quality of life was measured using the total score of the Quality of Life in Alzheimer's Disease (QoL-AD) questionnaire [20], which is a 13-item measure with a 4-point Likert scale. The EuroQoL-5D-3L [21] (EQ5D) questionnaire was also administered, which is a self-completion questionnaire that consists of 5 questions along with a scale for the participant to rate their health state on a scale thermometer of 0 to 100 (EQ-VAS). The European value set of Köning et al [22] was used for calculation of the EQ5D score.

Functional decline was assessed with the Lawton Instrumental Activities of Daily Living (IADL) [23] scale in people with dementia/mild cognitive impairment. The IADL uses 8 items for women and 5 items for men. The score was rescaled to range between 0 and 1.

The severity of cognitive impairment was assessed with the MMSE [24]. Depression was scored with the GDS-15 [25], which is a widely used scale to assess geriatric depression with 15 items and a range of 0-4 (normal), 5-8 (mildly depressed), 9-11 (moderately depressed), and 12-15 (severely depressed). Since an inclusion criterion for study participation was a GDS-15 score<11, the range in this study was 0–11.

Sociodemographic data included age, gender, education, living arrangement, marital status, and country of residence. Medical history included a diagnosis of dementia and comorbidity based on the International Classification of Diseases-10 [26].

#### Independent Variables for Caregivers

The independent variables for caregivers included the caregiver burden, which was assessed using the Zarit Caregiver Burden Interview (ZBI-12) [27,28], a 12-item 5-point Likert scale questionnaire. This is the short version of the original scale and was designed specifically for caregivers of people with dementia/mild cognitive impairment. Health-related quality of life was measured using the EQ5D as described above. The sociodemographic data included age, gender, education, living arrangements, marital status, country of residence, and relationship with the patient.

### Statistical Analysis

The mean (SD) and frequency and percentages are used to describe continuous and categorical variables, respectively. To compare groups with high and low technophilia, we used the median TechPh Index (2.83 in people with dementia/mild cognitive impairment and 3.00 in caregivers) as the cut-off point. To compare groups, we used the Chi square test for categorical variables and the Mann-Whitney test for continuous variables. A multivariate linear regression analysis was conducted to analyze the factors associated with technophilia. To check the assumptions of the linear model, the Breusch-Pagan test (homoscedasticity), Shapiro-Wilk normality test of the residuals, variance inflation factor (VIF; multicollinearity), and a scatter plot for the linearity of the variables were used. We also analyzed the factors associated with the use of specific apps or software to support memory using multivariate logistic regression. To check the goodness of fit, we used the Hosmer-Lemeshow test and the VIF for multicollinearity. If the VIF was higher than 2, the variable was taken out of the model. A 95% level of significance was used for assessment. R program version 3.6.1 (R Foundation for Statistical Computing, Vienna, Austria) and the R Commander package were used for these analyses.

## Results

The basic characteristics of the sample of people with dementia/mild cognitive impairment are summarized in Table 1. Nearly a third of the sample were diagnosed with dementia. A total of 588 of the 1086 people with dementia/mild cognitive impairment (54.14%) used smartphones and tablets almost every day and 284 (26.15%) had never used these technologies. Only 381/1086 (35.08%) used the internet on smartphones and tablets almost every day and 470/1086 (43.28%) never used the internet on these gadgets. More than half (706/1086, 65.01%) considered themselves not at all or quite knowledgeable when it comes to using a smartphone or a tablet. A total of 106 (9.76%) had a special app or software on their mobile phone or tablet to support memory, and 669/1086 (61.60%) believed that using a mobile phone or tablet to support their memory helps them remember things. Additional information about the use of smartphones and tablets by the people with dementia/mild cognitive impairment is provided in Multimedia Appendix 1. Information about groups with high and low technophilia and groups that use or do not use specific apps or software on their mobile phone or tablet to support memory is shown in Table 1.

**Table 1:** Characteristics of participants with dementia or mild cognitive impairment.

Variables		All participants (N=1086)^a^	High technophilia (N=591)^b^	Low technophilia (N=493)^c^	*P* value	Use apps to support memory (N=106)^d^	Do not use apps to support memory (N=877)^e^	*P* value
Age (years), mean (SD)		74.48 (7.24)	74.32 (7.32)	74.57 (7.16)	.72	70.22 (7.46)	74.76 (7.11)	<.001
**Gender, n (%)**					<.001			.29
	Female	576 (53.0)	276 (46.7)	298 (60.4)		51 (48.1)	470 (53.6)	
	Male	510 (47.0)	315 (53.3)	195 (39.6)		55 (51.9)	407 (46.4)	
**Education level, n (%)**					.003			.03
	Elementary school	653 (60.4)	331 (56.3)	320 (65.2)		55 (51.9)	527 (60.4)	
	Secondary school	225 (20.8)	126 (21.4)	99 (20.2)		21 (19.8)	190 (21.8)	
	Higher education	203 (18.8)	131 (22.2)	72 (14.7)		30 (28.3)	155 (17.8)	
**Marital status, n (%)**					.06			.06
	Unmarried	41 (3.8)	20 (3.4)	21 (4.3)		6 (5.7)	31 (3.5)	
	Married	697 (64.2)	377 (43.9)	320 (64.9)		75 (70.8)	555 (63.4)	
	Common law partner	44 (4.1)	33 (5.6)	11 (2.2)		7 (6.6)	35 (4.0)	
	Divorced	58 (5.3)	34 (5.8)	24 (4.9)		5 (4.7)	49 (5.6)	
	Widowed	245 (22.6)	126 (21.4)	117 (23.7)		13 (12.3)	206 (23.5)	
**Living arrangement, n (%)**					.22			.05
	Single	222 (20.6)	109 (18.6)	113 (23.1)		19 (17.9)	177 (20.4)	
	Spouse/partner	686 (63.6)	385 (65.7)	301 (61.6)		73 (68.9)	549 (63.2)	
	Children	97 (9.0)	57 (9.7)	40 (8.2)		3 (2.8)	85 (9.8)	
	Other	73 (6.8)	36 (6.1)	35 (7.2)		11 (10.4)	58 (6.7)	
**Country of residence, n (%)**					<.001			.12
	Sweden	345 (31.8)	231 (39.1)	114 (23.1)		39 (36.8)	258 (29.4)	
	Spain	741 (68.2)	360 (60.9)	379 (76.9)		67 (63.2)	619 (70.6)	
**Diagnosis of dementia, n (%)**					.73			.007
	Yes	299 (28.5)	165 (28.8)	132 (27.8)		87 (82.9)	594 (70.2)	
	No	750 (71.5)	408 (71.2)	342 (72.2)		18 (17.1)	252 (29.8)	
**Diagnosis of other medical condition, n (%)**					.04			.96
	Yes	267 (24.6)	431 (72.9)	386 (78.3)		26 (24.5)	213 (24.3)	
	No	819 (75.4)	160 (27.1)	107 (21.7)		80 (75.5)	664 (75.7)	
Cognitive Status (MMSE^f^), mean (SD)		25.41 (2.48)	25.51 (2.53)	25.32 (2.40)	.05	25.97 (2.05)	25.30 (2.56)	.03
Depression (GDS-15^g^), mean (SD)		3.03 (2.84)	2.47 (2.53)	3.70 (3.05)	<.001	3.06 (3.01)	3.11 (2.84)	.53
Instrumental activities (IADL^h^), mean (SD)		0.87 (0.19)	0.87 (0.20)	0.86 (0.19)	.07	0.91 (0.17)	0.86 (0.19)	.008
Quality of life (QoL-AD^i^), mean (SD)		36.08 (6.52)	37.67 (6.31)	34.21 (6.27)	<.001	36.93 (7.32)	36.00 (6.49)	.21
Quality of life (EQ5D^j^), mean (SD)		0.75 (0.22)	0.78 (0.22)	0.72 (0.22)	<.001	0.69 (0.25)	0.75 (0.22)	.08
Health State thermometer, mean (SD)		69.45 (19.60)	72.98 (18.70)	65.27 (19.88)	<.001	70.67 (19.03)	69.22 (19.80)	.43

^a^Education level N=1081; Living arrangement N=1078; Marital status N=1085; Diagnosis of dementia N=1049.

^b^Education level N=588; Marital status N=590; Living arrangement N=586; Diagnosis of dementia N=573.

^c^Education level N=491; Living arrangement N=489; Diagnosis of dementia N=474.

^d^Diagnosis of dementia N=105.

^e^Education level N=872; Marital status N=876; Living arrangement N=869; Diagnosis of dementia N=846.

^f^MMSE: Mini Mental State Examination.

^g^GDS-15: Geriatric Depression Scale.

^h^IADL: Lawton Instrumental Activities of Daily Living.

^i^QoL-AD: Quality of Life in Alzheimer’s Disease.

^j^EQ5D: EuroQoL-5D-3L.

The basic characteristics of the caregivers are summarized in Table 2. Among the 1086 caregivers, 839 (77.26%) used touchscreen technologies almost every day, and 123 (11.33%) had never used smartphones or tablets. A total of 721 (66.39%) used the internet on smartphones and tablets almost every day, and 191 (17.59%) never used the internet on touchscreen gadgets. A total of 433 (39.87%) considered themselves not at all or quite knowledgeable when it comes to using a smartphone or a tablet. A total of 181 (16.67%) had a special app or software on their mobile phone or tablet to support their memory, and 773 (71.18%) believed that using a mobile phone or tablet to support their memory helps them to remember things. Additional information about the use of smartphones and tablets by the caregivers is provided in Multimedia Appendix 1. Information about the caregivers overall, groups with high and low technophilia, and groups that use or do not use specific apps or software on their tablet or mobile phone to support memory is shown in Table 2.

**Table 2:** Characteristics of caregivers.

Variables		Total (N=1086)^a^	High technophilia (N=464)^b^	Low technophilia (N=598)^c^	*P* value	Use apps to support memory (N=181)^d^	Do not use apps to support memory (N=840)^e^	*P* value
Age (years), mean (SD)		62.29 (14.68)	58.35 (15.84)	65.24 (12.95)	<.001	54.27 (14.49)	63.34 (14.20)	<.001
**Gender, n (%)**					.003			.11
	Female	741 (68.2)	295 (63.6)	167 (27.9)		133 (73.5)	566 (67.4)	
	Male	345 (31.8)	169 (36.4)	431 (72.1)		48 (26.5)	274 (32.6)	
**Education level, n (%)**					<.001			<.001
	Elementary school	378 (35.7)	111 (24.3)	257 (44.2)		29 (16.2)	318 (38.8)	
	Secondary school	345 (32.5)	167 (36.6)	173 (29.3)		73 (40.8)	256 (31.3)	
	Higher education	337 (31.8)	178 (39.0)	152 (26.1)		77 (43.0)	245 (29.9)	
**Marital status, n (%)**					<.001			.005
	Unmarried	126 (11.6)	75 (16.2)	46 (7.7)		33 (18.2)	88 (10.5)	
	Married	804 (74.2)	312 (67.4)	475 (79.6)		121 (66.9)	633 (75.5)	
	Common law partner	76 (7.0)	38 (8.2)	37 (6.2)		15 (8.3)	55 (6.6)	
	Divorced	47 (4.3)	23 (5.0)	24 (4.0)		11 (6.1)	34 (4.1)	
	Widowed	30 (2.8)	15 (3.2)	15 (2.5)		1 (0.5)	28 (3.3)	
**Living arrangement, n (%)**					.005			.30
	Single	98 (9.0)	50 (10.8)	45 (7.5)		17 (9.4)	75 (8.9)	
	Spouse/partner	738 (68.1)	291 (62.9)	436 (73.0)		112 (61.9)	576 (68.7)	
	Children	100 (9.2)	52 (11.2)	45 (7.5)		21 (11.6)	77 (9.2)	
	Other	147 (13.6)	70 (15.1)	71 (11.9)		31 (17.1)	110 (13.1)	
**Country of residence, n (%)**					.03			.05
	Sweden	345 (31.8)	167 (36.0)	177 (29.6)		44 (24.3)	266 (31.7)	
	Spain	741 (68.2)	297 (64.0)	421 (70.4)		137 (75.7)	574 (68.3)	
**Relation with the patient, n (%)**					<.001			<.001
	Spouse/partner	590 (55.5)	204 (44.7)	374 (63.9)		59 (33.7)	483 (58.6)	
	Child	320 (30.1)	179 (39.3)	134 (22.9)		97 (55.4)	214 (26.0)	
	Other	153 (14.4)	73 (16.0)	77 (13.2)		19 (10.9)	127 (15.4)	
Caregiver Burden (ZBI-12^f^), mean (SD)		6.86 (7.70)	6.31 (7.21)	7.28 (8.08)	.13	9.02 (7.62)	6.53 (7.68)	<.001
Quality of life (EQ5D^g^), mean (SD)		0.77 (0.21)	0.81 (0.20)	0.75 (0.22)	<.001	0.77 (0.22)	0.78 (0.21)	.72
Health State (thermometer), mean (SD)		72.24 (18.62)	74.95 (17.36)	70.07 (19.22)	<.001	70.94 (18.21)	72.46 (18.67)	.22

^a^Education level N=1060; Marital status N=1083; Living arrangement N=1083; Relation with the patient N=1063.

^b^Education level N=456; Marital status N=463; Living arrangement N=463; Relation with the patient N=456.

^c^Education level N=582; Marital status N=597; Living arrangement N=597; Relation with the patient N=585.

^d^Education level N=179; Relation with the patient N=175.

^e^Education level N=819; Marital status N=838; Living arrangement N=838; Relation with the patient N=824.

^f^ZBI-12: Zarit Caregiver Burden Interview.

^g^EQ5D: EuroQoL-5D-3L.

The people with dementia/mild cognitive impairment had a mean TechPH index of 2.84 (SD 0.69). The TechEnthusiasm score was 2.95 (SD 1.07) and the TechAnxiety score was 3.30 (SD 0.95). The caregivers had a TechPH index score of 3.07 (SD 0.68); the TechEnthusiasm score was 3.31 (SD 1.00) and the TechAnxiety score was 3.19 (SD 0.95). There were differences in the TechPH index between patients and caregivers (B=0.223, SE 0.030, *P*<.001), which remained significant after adjusting by age, gender, education level, and health status (B=0.074, SE 0.032, *P*=.02). There were significant differences in TechAnxiety between patients and caregivers (B=–0.120, SE 0.041, *P*=0.004); however, these differences were not significant after adjusting for age, gender, education level, and health status (B=–0.042, SE 0.047, *P*=.37). There was a significant difference in TechEnthusiasm between patients and caregivers (B=0.360, SE 0.045, *P*<.001), and this difference was still significant after adjusting for confounders (B=0.128, SE 0.050, *P*=.01). There were significant differences in the use of specifics apps or software to support memory between patients and caregivers (odds ratio [OR]=1.783, SE 0.131, *P*<.001); however, these differences were not significant after adjusting for confounders (OR=0.818, SE 0.167, *P*=.23).

In the multivariate analysis, the TechPH index in people with dementia/mild cognitive impairment was related to lower age, male gender, higher education level, living arrangement (living with children vs single), country of residence (Sweden vs Spain), depression, and health status (Table 3). In caregivers, the TechPH index was associated with lower age, male gender, higher education level, country of residence (Sweden vs Spain), lower caregiver burden, and better quality of life (Table 4). The use of specific apps or software on tablets or mobile phones to support memory was only associated with age in people with dementia/mild cognitive impairment (Table 5) and was associated with age, education level, and caregiver burden in caregivers (Table 6).

**Table 3:** Multivariate linear regression model^a^ of factors associated with technophilia in people with dementia/mild cognitive impairment.

Variables		Estimate	SE	t_1008_	*P* value	VIF^b^
Intercept		3.763	0.402	9.359	<.001	
Age		–0.009	0.003	–2.850	.004	1.248
Gender: female (male=reference)		–0.160	0.044	–3.630	<.001	1.211
**Education level (elementary school=reference)**					.01	1.252
	Secondary school	0.021	0.052	0.400	.69	
	Higher education	0.167	0.058	2.886	.004	
**Living arrangement (children=reference)**					.01	1.342
	Single	–0.210	0.083	–2.538	.01	
	Spouse/partner	–0.149	0.074	–1.999	.05	
	Other	–0.137	0.103	–1.328	.18	
Country: Sweden (Spain=reference)		0.256	0.058	4.414	<.001	1.855
Diagnosis of dementia: Yes (no=reference)		0.060	0.054	1.116	.26	1.483
Diagnosis of other medical condition: Yes (no=reference)		0.047	0.050	0.939	.35	1.180
Cognitive Status (MMSE^c^)		–0.013	0.010	–1.356	.17	1.438
Depression (GDS-15^d^)		–0.046	0.008	–5.625	<.001	1.360
Instrumental activities (IADL^e^)		–0.020	0.125	–0.164	.87	1.450
Health State (Thermometer)		0.004	0.001	3.570	<.001	1.271

^a^Breusch-Pagan test *P*=.06; Shapiro-Wilk normality test of the residuals *P*=0.39; adjusted *R^2^*=0.146.

^b^VIF: variance inflation factor.

^c^MMSE: Mini Mental State Examination.

^d^GDS-15: Geriatric Depression Scale.

^e^IADL: Lawton Instrumental Activities of Daily Living.

**Table 4:** Multivariate linear regression model^a^ of factors associated with technophilia in caregivers.

Variables		Estimate	SE	t_1021_	*P* value	VIF^b^
Intercept		3.824	0.159	24.014	<.001	
Age		–0.016	0.002	–9.560	<.001	1.603
Female gender (male=reference)		–0.191	0.043	–4.470	<.001	1.081
**Education level (elementary school=reference)**					<.001	1.278
	Secondary school	0.084	0.050	1.677	.01	
	Higher education	0.201	0.052	3.879	<.001	
**Living arrangement (children=reference)**					.27	1.379
	Single	0.050	0.092	0.537	.59	
	Spouse/partner	–0.062	0.074	–0.843	.40	
	Other	–0.094	0.083	–1.128	.26	
Country: Sweden (Spain=reference)		0.245	0.048	5.140	<.001	1.375
Caregiver Burden (ZBI-12^c^)		–0.005	0.003	–2.055	.04	1.154
Quality of life (EQ5D^d^)		0.348	0.097	3.592	<.001	1.165

^a^Breusch-Pagan test *P*=.07; Shapiro-Wilk normality test of the residuals *P*=0.84; adjusted *R^2^*=0.194.

^b^VIF: variance inflation factor.

^c^ZBI-12: Zarit Caregiver Burden Interview.

^d^EQ5D: EuroQoL-5D-3L.

**Table 5:** Multivariate logistic regression model^a^ of factors associated with use of apps or specific software to support memory in people with dementia/mild cognitive impairment.

Variables		Estimate	SE	*z*	*P* value	OR^b^	95% CI	VIF^c^
Intercept		3.189	2.247	1.419	.16			
Age		–0.093	0.017	–5.387	<.001	0.911	0.880-0.942	1.366
Gender: female (male=reference)		–0.119	0.233	–0.512	.61	0.877	0.562-1.402	1.183
**Education level (elementary school= reference)**					.08			1.362
	Secondary school	–0.145	0.292	–0.496	.62	0.865	0.488-1.534	
	Higher education	0.548	0.290	1.886	.06	1.729	0.979-3.056	
**Living arrangement (children=reference)**					.16			1.357
	Single	1.207	0.663	1.822	.07	3.345	0.912-12.262	
	Spouse/partner	1.090	0.625	1.745	.08	2.975	0.874-10.121	
	Other	1.347	0.699	1.926	.05	3.844	0.976-15.139	
Country: Sweden (Spain=reference)		0.319	0.312	1.019	.31	1.375	0.745-2.538	1.990
Diagnosis of dementia: Yes (No=reference)		–0.391	0.330	–1.187	.23	0.676	0.354-1.290	1.431
Diagnosis of other medical condition: Yes (No=reference)		0.168	0.262	0.642	.52	1.183	0.708-1.977	1.132
Cognitive Status (MMSE^d^)		–0.001	0.056	–0.022	.98	0.999	0.894-1.115	1.373
Depression (GDS-15^e^)		–0.008	0.047	–0.165	.87	0.992	0.906-1.087	1.565
Instrumental activities (IADL^f^)		0.165	0.744	0.222	.82	1.180	0.274-5.074	1.360
Health State (Thermometer)		0.002	0.006	0.367	.71	1.002	0.990-1.015	1.411

^a^Hosmer and Lemeshow goodness of fit test *P*=.23.

^b^OR: odds ratio.

^c^VIF: variance inflation factor.

^d^MMSE: Mini Mental State Examination.

^e^GDS-15: Geriatric Depression Scale.

^f^IADL: Lawton Instrumental Activities of Daily Living.

**Table 6:** Multivariate logistic regression model^a^ of factors associated with use of apps or specific software to support memory in caregivers.

Variables		Estimate	SE	*z*	*P* value	OR^b^	95% CI	VIF^c^
Intercept		0.089	0.683	0.131	.90			
Age		–0.038	0.007	-5.441	<.001	0.962	0.949-0.976	1.519
Female gender (male=reference)		0.069	0.199	0.346	.73	1.072	0.725-1.585	1.068
**Education level (elementary school=reference)**					<.001			1.180
	Secondary school	0.828	0.252	3.279	.001	2.289	1.395-3.754	
	Higher education	0.960	0.257	3.738	<.001	2.611	1.579-4.319	
**Living arrangement (children=reference)**					.43			1.375
	Single	0.080	0.386	0.208	.83	1.083	0.508-2.311	
	Spouse/partner	0.332	0.298	1.113	.27	1.394	0.777-2.502	
	Other	–0.052	0.337	–0.153	.88	0.950	0.491-1.838	
Country: Sweden (Spain=reference)		–0.126	0.225	–0.561	.57	0.881	0.567-1.370	1.327
Caregiver Burden (ZBI-12^d^)		0.023	0.011	2.084	.04	1.023	1.001-1.046	1.141
Quality of life (EQ5D^e^)		–0.540	0.429	–1.259	.21	0.582	0.251-1.351	1.194

^a^Hosmer and Lemeshow goodness of fit test *P*=.68.

^b^OR: odds ratio.

^c^VIF: variance inflation factor.

^d^ZBI-12: Zarit Caregiver Burden Interview.

^e^EQ5D: EuroQoL-5D-3L.

## Discussion

In people with dementia/mild cognitive impairment, technophilia was associated with less depression, better health status, as well as with sociodemographic variables. In caregivers, technophilia was associated with a better quality of life, less care burden, and other sociodemographic variables. The results of this study indicate that people with dementia/mild cognitive impairment have less technophilia than their caregivers, specifically less TechEnthusiasm, but no differences were found in TechAnxiety. The people with dementia/mild cognitive impairment used smartphones and tablets with specific apps or software to support their memory less than their caregivers, despite being a population who would benefit most from these apps. These differences are likely due to age barriers.

The use of smartphones and tablets in our study was lower than that reported in a recent study in Australia in which 91.4% of people with dementia/mild cognitive impairment reported routinely using smartphones [29]. Likewise, in other studies that included older adults and populations with cognitive impairment and dementia, attitudes toward technology were associated with factors such as age [30-32], male gender [33], higher education level [29,30,33,34], depression and negative cognitions [35], and health status [30], and variability was found between countries [36]. A correlation between the use of health apps and age has also been reported [32,37]. However, some results in other studies have not been replicated. For example, one study reported an association between cognitive functioning and the use of technologies in a cognitively impaired population [38], and another study found that women used more health apps than men [39].

The differences in technophilia between people with dementia/mild cognitive impairment and caregivers, mainly due to the lack of technology enthusiasm, could be related to dementia or mild cognitive impairment itself. In fact, other studies have found that dementia and mild cognitive impairment were related to more perceived difficulties in everyday technology use [40]. In addition, this lack of enthusiasm for technology could be related to the apathy associated with dementia and mild cognitive impairment, which is a persistent behavioral symptom [41,42]. This apathy could affect the enthusiasm toward new technologies and may be a barrier for interventions that demand learning and adaptation to the use of these technologies.

Other studies have also found that barriers to the use of ICTs and assistive technologies in older adults are due to the lack of “interest or relevance to life” and the perception of “no need” [15,43]. Indeed, these ICTs are often not designed specifically to cover the real needs of this population and they do not arouse interest. Other studies have also found that a lack of knowledge in older adults is a barrier to the use of technologies [11,16]. In addition, age was identified as one of the most critical determinants of the use of apps to support memory after adjusting for health status. This result coincides with other studies [15,30] in the general population.

Interestingly, technophilia was also strongly associated with health status and depression after adjusting for possible confounders such as gender, age, education level, and cognitive function. One longitudinal study conducted by Cotten et al [44] and other studies [45,46] have found that use of the internet and ICTs in older individuals is associated with less depression and a better quality of life, and there is some evidence that this association is mediated by loneliness and social isolation [44,45]. In conclusion, these results highlight the potential barriers of interventions for the use of ICTs, including smartphones and tablets, to improve the quality of life of people with dementia/mild cognitive impairment. The nature of the relation between use and technophilia and quality of life and mood needs to be clarified with longitudinal and experimental studies.

The use of specific apps or software to support memory was not associated with better cognitive function, better quality of life, or less depression. However, some positive results have emerged from several meta-analyses reporting that computerized cognitive training and electronic health apps are effective in improving cognition and quality of life in people with dementia/mild cognitive impairment [47,48]. This result comes from formal interventions, and the results of the present study could be due to possible confounders not assessed in the study or because the participants were not using the apps or software adequately (eg, insufficient training time or not using well-designed apps). Other studies have also indicated inconsistencies and lack evidence regarding the effectiveness of ICT interventions to improve cognition and other health-related variables [49,50].

The results of this study need to be considered in the context of several limitations. This was a cross-sectional study, and therefore the causal relation of the variables could not be established. More longitudinal and interventional studies are necessary to determine the effects of ICT use and the attitudes toward technology in people with dementia/mild cognitive impairment and their caregivers and to identify the factors that influence these variables in the other direction. In addition, this was a secondary analysis from a clinical trial, and the sample may not be representative of all people with dementia/mild cognitive impairment and caregivers. Individuals with severe depression were excluded and more than half of the sample was from only one country. There was no established cut-off point for the definition of high or low technophilia, which limits the use of the mean as the cutoff. Another limitation is that only a small set of variables was assessed. However, the strength of the study is the large sample used with more than 2000 individuals.

In conclusion, the factors associated with technophilia suggest potential barriers to technological interventions in people with dementia/mild cognitive impairment and their caregivers. These results have implications on the possible usefulness of considering technophilia and enthusiasm toward ICTs as determinants and moderator elements in digital interventions in the elderly population. The results suggest that designers of apps for older adults with cognitive impairment should create apps that engage users and are designed specifically for their needs as this population frequently lacks enthusiasm for technology. The use of touchscreen technologies was also frequent among people with dementia/mild cognitive impairment, whereas the use of specific apps or software to support memory was reduced in people with dementia/mild cognitive impairment and caregivers, and they face age barriers for the use of these apps. However, the majority of this population considers that use of smartphones or tablets is helpful for memory, which highlights the presence of a gap between the perceived potential and actual use of these technologies.

## Appendix

Multimedia Appendix 1 Detailed data on use of smartphones and tablets by people with dementia/mild cognitive impairment (PwD/MCI).

## Abbreviations

EQ5D: EuroQoL-5D-3L
EQ-VAS: Scale thermometer of 0-100
GDS-15: Geriatric Depression Scale
IADL: Lawton Instrumental Activities of Daily Living
ICT: information and communication technology
MMSE: Mini-Mental State Examination
OR: odds ratio
QoL-AD: Quality of Life in Alzheimer's Disease
SMART4MD: Support Monitoring and Reminder for Mild Dementia
VIF: variance inflation factor
ZBI-12: Zarit Caregiver Burden Interview
