# Supplementary material for: Attitudes and Use of Information and Communication Technologies in Older Adults With Mild Cognitive Impairment or Early Stages of Dementia and Their Caregivers: Cross-Sectional Study
Source: J Med Internet Res. 2020 Jun 1;22(6):e17253. doi: 10.2196/17253 (PMC7296403; doi:10.2196/17253)
Supplement: Multimedia Appendix 1 [file jmir_v22i6e17253_app1.docx]

## Appendix

588 of PwD/MCI (54.14% ) used smartphones and tablets almost every day, 95 (8.74%) at least once a week but not every day, 30 (2.76%) less than once a week, 83 (7.64%) not at all, and 284 (26.15%) had never used these technologies (6, 0.55% no answer). Only 381 (35.08%) used internet in smartphones and tablets almost every day, 94 (8.66%) at least once a week but not every day, 62 (5.71%) less than once a week and 470 (43.28%) never used internet in these gadgets (79, 7.27% no answer). More than half (N=706; 65.01%) considered themselves not at all or quite knowledgeable when it comes to using a smartphone or a tablet. 207 (19.06 %) used the mobile phone or tablet as a way to support their memory and 106 (9.76%) had a special app or software on their mobile phone or tablet to support memory. 669 (61.16%) believed that using a mobile phone or tablet to support their memory helps them remember things.

839 (77.26%) of caregivers used touchscreen technologies almost every day, 52 (4.79%) at least once a week, 15 (1.38%) less than once a week, 33 (3.04%) not at all and 123 (11.33%) had never used smartphones or tablets (24, 2.21% no answer). 721 (66.39%) used internet in smartphones and tablets almost every day, 86 (7.92%) at least once a week, 32 (2.95%) less than once a week and, 191 (17.59%) never used internet in touchscreen gadgets (79, 7.27% no answer). 433 (39.87%) considered themselves not at all or quite knowledgeable when it comes to using a smartphone or a tablet. 359 (33.05 %) used the mobile phone or tablet as a way to support memory and 181 (16.67%) had a special app or software on their mobile phone or tablet to support their memory. 773 (71.18%) believed that using a mobile phone or tablet to support their memory helps them remember things.
